# *PTPN11* Mosaicism Causes a Spectrum of Pigmentary and Vascular Neurocutaneous Disorders and Predisposes to Melanoma

**DOI:** 10.1016/j.jid.2022.09.661

**Published:** 2023-06

**Authors:** Satyamaanasa Polubothu, Nicole Bender, Siobhan Muthiah, Davide Zecchin, Charalambos Demetriou, Sara Barberan Martin, Sony Malhotra, Jana Travnickova, Zhiqiang Zeng, Markus Böhm, Sebastien Barbarot, Catherine Cottrell, Olivia Davies, Eulalia Baselga, Nigel P. Burrows, Virginie Carmignac, Joey Santiago Diaz, Christine Fink, Holger A. Haenssle, Rudolf Happle, Mark Harland, Jacquelyn Majerowski, Pierre Vabres, Marie Vincent, Julia A. Newton-Bishop, D. Tim Bishop, Dawn Siegel, E. Elizabeth Patton, Maya Topf, Neil Rajan, Beth Drolet, Veronica A. Kinsler

**Affiliations:** 1 Mosaicism and Precision Medicine Laboratory, The Francis Crick Institute, London, United Kingdom; 2 Paediatric Dermatology, Great Ormond Street Hospital, London, United Kingdom; 3 Genetics and Genomic Medicine, UCL Great Ormond Street Institute of Child Health, London, United Kingdom; 4 Department of Dermatology, Medical College of Wisconsin, Milwaukee, Wisconsin, USA; 5 Institute of Genetic Medicine, Newcastle University, Newcastle upon Tyne, United Kingdom; 6 Scientific Computing Department, Science and Technology Facilities Council, Research Complex at Harwell, Harwell Oxford, United Kingdom; 7 MRC Human Genetics Unit and Cancer Research UK Edinburgh Centre, MRC Institute of Genetics & Molecular Medicine, University of Edinburgh, Edinburgh, United Kingdom; 8 Department of Dermatology, University of Münster, Münster, Germany; 9 Department of Dermatology, Centre Hospitalier Universitaire Nantes, Nantes, France; 10 Institute for Genomic Medicine, Nationwide Childrens’ Hospital, Columbus, USA; 11 Department of Dermatology, SJD Barcelona Children's Hospital, Barcelona, Spain; 12 Department of Dermatology, Addenbrooke’s Hospital, Cambridge, United Kingdom; 13 Génétique des Anomalies du Développement, Université de Bourgogne, Dijon, France; 14 Section of Epidemiology and Biostatistics, Leeds Institute of Cancer and Pathology, Cancer Research UK Clinical Centre at Leeds, St James's University Hospital, Leeds, United Kingdom; 15 Department of Statistics, College of Science, Central Luzon State University, Science City of Munoz, Philippines; 16 Department of Physical Sciences and Mathematics, College of Arts and Sciences, University of the Philippines Manila Ermita, Manila, Philippines; 17 Department of Dermatology, University of Heidelberg, Heidelberg, Germany; 18 Department of Dermatology, Medical Center, University of Freiburg, Freiburg, Germany; 19 Department of Dermatology, CHU Dijon, Dijon, France; 20 Division of Haematology and Immunology, Leeds Institute of Medical Research, Leeds, United Kingdom; 21 Centre for Structural Systems Biology, Leibniz-Institut für Virologie (LIV) and Universitätsklinikum Hamburg-Eppendorf (UKE), Hamburg, Germany

## Abstract

Phakomatosis pigmentovascularis is a diagnosis that denotes the coexistence of pigmentary and vascular birthmarks of specific types, accompanied by variable multisystem involvement, including CNS disease, asymmetrical growth, and a predisposition to malignancy. Using a tight phenotypic group and high-depth next-generation sequencing of affected tissues, we discover here clonal mosaic variants in gene *PTPN11* encoding SHP2 phosphatase as a cause of phakomatosis pigmentovascularis type III or spilorosea. Within an individual, the same variant is found in distinct pigmentary and vascular birthmarks and is undetectable in blood. We go on to show that the same variants can cause either the pigmentary or vascular phenotypes alone, and drive melanoma development within pigmentary lesions. Protein structure modeling highlights that although variants lead to loss of function at the level of the phosphatase domain, resultant conformational changes promote longer ligand binding. In vitro modeling of the missense variants confirms downstream MAPK pathway overactivation and widespread disruption of human endothelial cell angiogenesis. Importantly, patients with *PTPN11* mosaicism theoretically risk passing on the variant to their children as the germline RASopathy Noonan syndrome with lentigines. These findings improve our understanding of the pathogenesis and biology of nevus spilus and capillary malformation syndromes, paving the way for better clinical management.

## Introduction

Human mosaic disorders are caused by single-cell postzygotic variants in the developing embryo or fetus. In the light of new knowledge of the ubiquitous nature of de novo somatic variants both before and after birth (Ju et al., 2017; Martincorena and Campbell, 2015), a recent consensus has redefined a mosaic disease as “the coexistence of cells with at least two genotypes, at least one of which is pathological, by the time of birth, in an individual derived from a single zygote, and which leads to a disease phenotype” (Kinsler et al., 2020). Sequencing advances in the last decade have facilitated genetic discovery in these diseases, identifying established oncogenes as the cause in many cases (Al-Olabi et al., 2018b; Groesser et al., 2012; Lindhurst et al., 2011; Shirley et al., 2013). Although monogenic in etiology, the developmental timing of the variant and the cell type in which it occurs vary, vastly increasing the complexity of disease manifestations (Kinsler et al., 2020). This causes major practical difficulties with phenotypic definition compared to germline conditions and has led to the reclassification of many clinical diagnoses as phenotypic variations along a genetic disease spectrum rather than distinct diseases (Groesser et al., 2012; Keppler-Noreuil et al., 2015). These new insights into genotype‒phenotype interaction have however brought about two major shifts in the management of mosaic diseases. The first is the implementation of targeted pharmacologic therapies (Keppler-Noreuil et al., 2019; Riachi et al., 2021; Venot et al., 2018), and the second is the increasing awareness of the possibility of passing on mosaic variants as germline conditions in the offspring (Kinsler et al., 2020).

**Figure 1:**
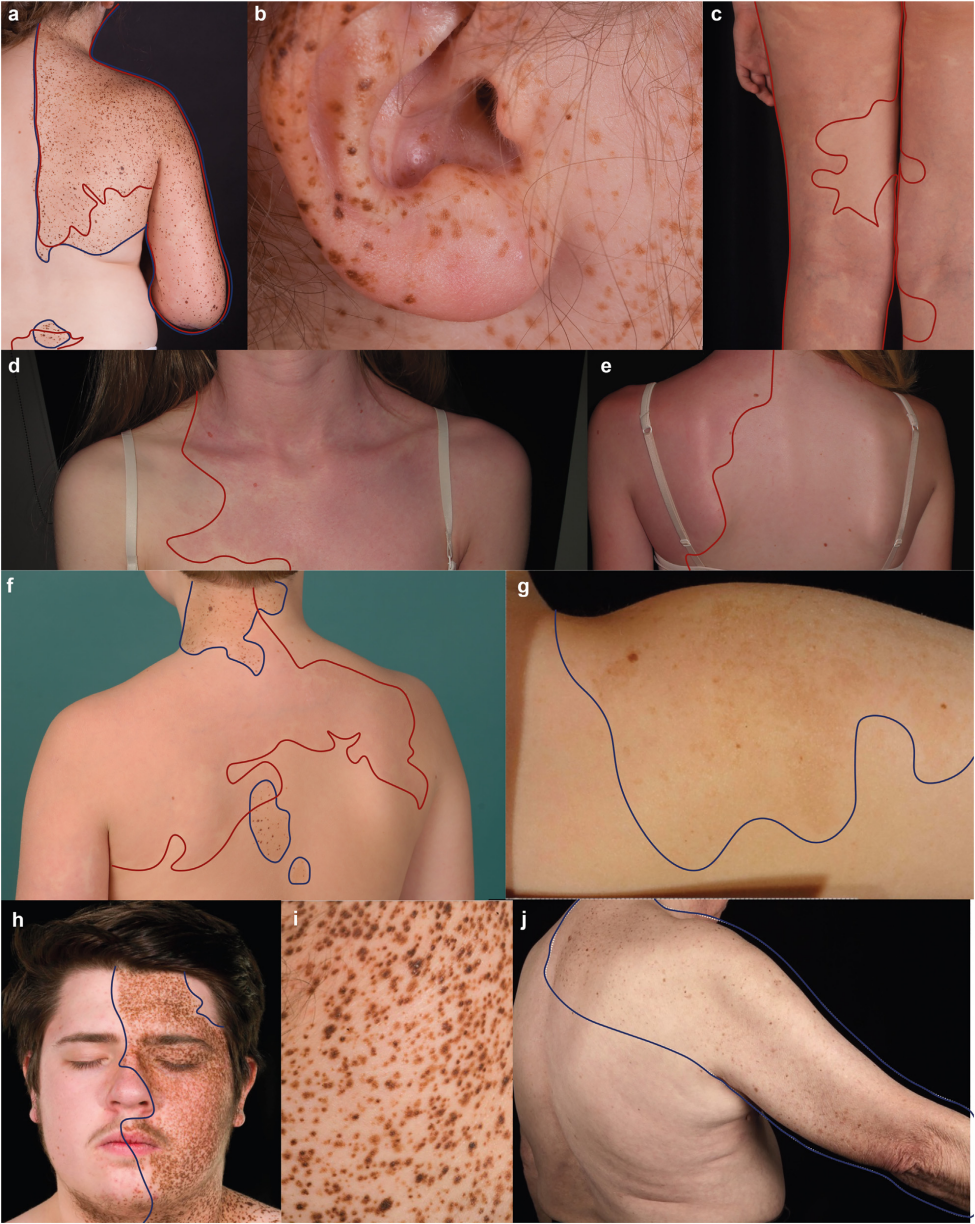
**Clinical images illustrating the broad spectrum of dermatological phenotypes caused by *PTPN11* mosaicism.** See Table 1 for the noncutaneous aspects of disease phenotypes. Of note, both macular and papular nevus spilus are seen, with substantial variation between patients. (**a‒c**) Co-occurrence of some separate and some overlapping pigmentary (nevus spilus) and vascular (capillary malformation) birthmarks in a case of PPV spilorosea. (**d‒e**) Capillary malformation without pigmentary abnormalities. (**f**) PPV spilorosea showing juxtaposition and some degree of overlap of both types of birthmarks. (**g**) Single nevus spilus associated with stage 4 neuroblastoma. (**h, i**) Facial and truncal nevus spilus in a segmental distribution without a vascular phenotype, and (**j**) mixed macular and papular nevus spilus in an older subject who developed two primary melanomas within the affected region. Pigmentary lesions are highlighted by a blue solid outline, and vascular lesions are highlighted by a red solid outline. Patients and/or parents/guardians provided written consent for the publication of images. PPV, phakomatosis pigmentovascularis. Patients and/or parents/guardians provided written consent for the publication of images.

With these potential benefits in mind, we set out to uncover the cellular and molecular basis of a distinct subtype of phakomatosis pigmentovascularis (PPV) type III (Ota et al., 1947) or spilorosea (Happle, 2005), a serious multisystem disease of unknown genetic etiology in which individuals are born with both speckled pigmented birthmarks (nevus spilus) and vascular birthmarks (capillary malformations) (Figure 1). Multiple associated abnormalities can coexist with the cutaneous findings, notably skeletal (scoliosis, limb asymmetry), neurological (neurodevelopmental delay, seizures, Moyamoya disease), and lymphoedema (Fink et al., 2016; Happle, 2005; Torchia, 2013; Tsuruta et al., 1999). There is also a risk of melanoma in nevus spilus in general (Borrego et al., 1994; Manganoni et al., 2012; Vidaurri-de la Cruz and Happle, 2006; Weinberg et al., 1998); however, the lack of genotypic diagnosis, the variability of nevus spilus phenotypes, and the absence of prospective cohort studies have so far not allowed this to be quantified or stratified. Importantly, it is not known whether PPV III/spilorosea can be passed on to future offspring and, if so, in what form. The phenotypes of our patients are detailed in Table 1. After genotypic discovery, all patients in the cohort were screened by echocardiography and electrocardiogram, and all were normal.

**Table 1:** Phenotypic and Genotypic Details of the Nine Patients Included in the Study

Patient (Age)	Clinical Diagnosis	Pigmentary Phenotype	Vascular Phenotype	Neurodevelopment	Musculoskeletal Phenotype	Other Phenotypes	Chromosomal Co-ordinates, cDNA and AA Change, Variant Allele Reads/Total Reads (% Allele Load); Transcript ENST00000351677
1 (10 y)	PPV III/spilorosea	Nevus spilus –multiple, nonsegmental, left neck, midline back, dorsum both feet	Capillary malformation on face, upper back bilaterally, left trunk, buttock, and leg	Developmental delay	Thoracic scoliosis convex to the right	Overgrowth of left leg and arm	Chr12:112926248G>A, c.1381G>A, p.(A461T) 212/1,715 Affected skin biopsy (12%) Blood (0%)
2 (11 y)	PPV III/spilorosea	Nevus spilus - single, segmental, unilateral, right sided, affecting the face, torso anterior and posterior, and arm	Capillary malformation on the face, trunk, right arm, and across both legs anterior and posterior	Moderate global developmental delay	Scoliosis convex to the left, muscular hypotonia of the right upper torso and face.	Macrocephaly, loss of visual acuity right eye (astigmatism), increased bilateral pigmentation of the ocular fundus.	Chr12:112926248G>A, c.1381G>A, p.(A461T) 972/7,111 Nevus spilus skin biopsy (14%)
3 (14 y)	Large segmental pattern nevus spilus	Nevus spilus – single, segmental, unilateral, clear midline cut-off left face, anterior/posterior upper chest extending onto the arm	N/A	Normal	Normal		Chr12:112926248G>A, c.1381G>A, p.(A461T) Nevus spilus skin biopsy 116/2,257 (5%)
4 (8 y)	PPV III/spilorosea	Nevus spilus - single, left lower abdomen	Capillary malformation – multiple, reticulate, dull pink, trunk, bilateral upper and lower extremities	Normal	Mild pectus excavatum		Chr12:112926248G>A, c.1381G>A, p.(A461T) Area of overlapping nevus spilus and capillary malformation (7.5%) Blood (0%)
5 (11 y)	Large single nevus spilus	Nevus spilus – no clear pattern, single, 10cm at age 6, left shoulder	N/A	Normal	Normal	Neuroblastoma	Chr12:112926270, c.1403C>T, p.(T468M) 342/19,991 Nevus spilus skin biopsy (2%)
6 (81 y)	Large segmental pattern nevus spilus and multiple primary melanomas within it	Nevus spilus – single, right upper posterior trunk extending full-length right arm	N/A	Normal	Normal	Multiple primary melanoma	Chr12:112926270, c.1403C>T, p.(T468M) Nevus spilus skin biopsy 103/2,597 (4%) Melanoma skin biopsy 744/1,249 (60%)
7 (15 y)	Large capillary malformation	N/A	Capillary malformation – single, left upper trunk anterior and posterior, extending to shoulder girdle and full-length left arm	Normal	Thoracic scoliosis convex to the right		Chr12: 112924336G>C, c.G1282C , p.(V428L) Capillary malformation skin biopsy 22.4%
8 (8 y)	Speckled lentiginous nevus syndrome	Nevus spilus – multiple, nonsegmental pattern, across midline over the lower back, also left knee and right forefoot	N/A	Normal	Lumbar kyphoscoliosis, abnormally low skeletal muscular tone, and power		Chr12:112888251-112927037, c.268_1599+58del, p.(E90-Q533del) Nevus spilus skin biopsy – accurate allele load unavailable as detected by Sanger sequencing Blood (0%)
9 (17 y)	PPV III/spilorosea	Nevus spilus	Capillary malformation	Not known	Not known	Woolly hair nevus, epidermal nevus	No causative variant detected in *PTPN11* on affected skin biopsy Blood (0%)

Abbreviations: AA, amino acid; N/A, not available; PPV, phakomatosis pigmentovascularis.

Note that the majority of patients have noncutaneous features of varying types, as would be expected in a mosaic disorder affecting an early cell type, where the protein is widely expressed. Patient 6 was confirmed as wild type for *NRAS* and *BRAF* variants in the melanoma samples.

## Results

**Figure 2:**
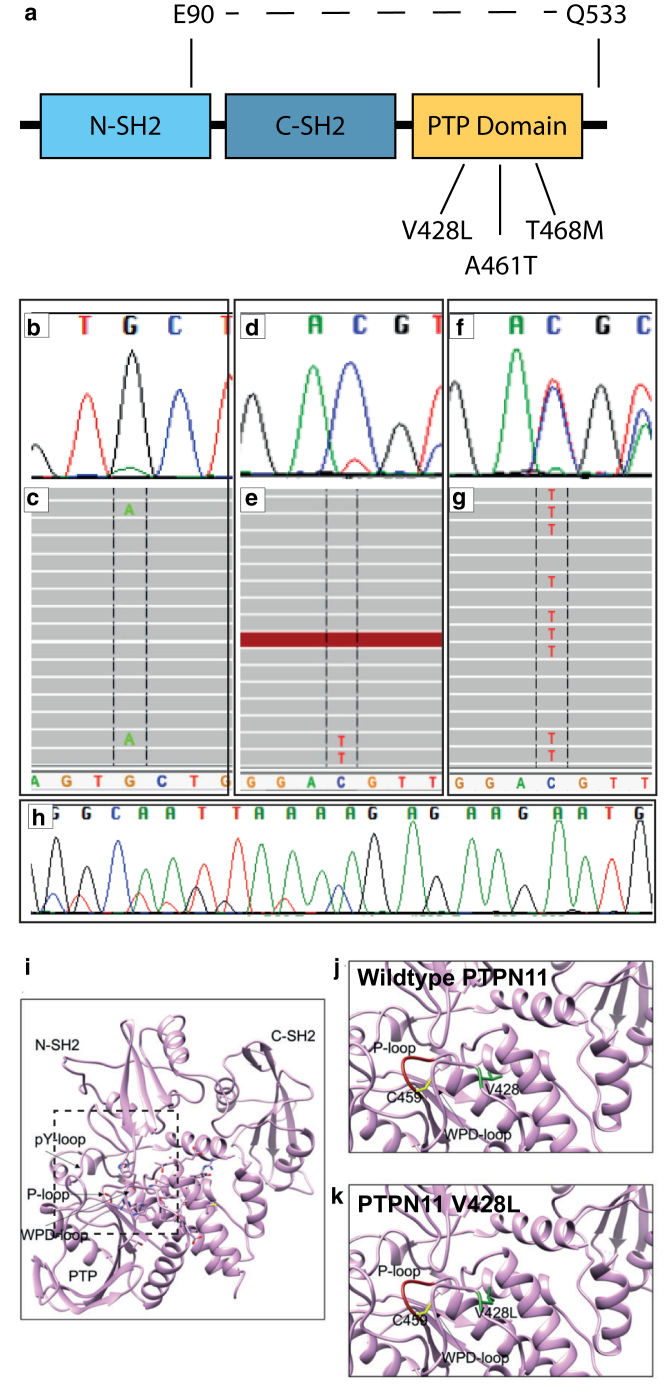
**Clonal *PTPN11* variants are present at mosaic levels in affected tissues and amplified in melanoma.** (**a**) Schematic representation of the functional domains in the *PTPN11* gene, indicating the three missense variants (V428L, A461T, T468M) and the large intragenic deletion (E90-Q533), all of which affect the PTP domain. (**b**) Chromatogram from Sanger sequencing of a nevus spilus and (**c**) NGS reads of the same sample represented in Integrated Genomics Viewer (Broad Institute), demonstrating mosaic variant in *PTPN11* c.1381G>A, p.(A461T); (**d**) nevus spilus chromatogram from Sanger sequencing and (**e**) NGS of the same sample demonstrating mosaic variant in *PTPN11* c.1403C>T, p.(T468M); (**f**) Sanger sequencing and (**g**) NGS showing heterozygous *PTPN11* c.1403C>T, p.(T468M) from a primary melanoma arising within a nevus spilus carrying the same variant. The allele load in the melanoma is much higher, indicating clonal expansion of PTPN11 variant melanocytes and therefore that the *PTPN11* variant is a driver. (**h**) Sanger sequencing showing the large intragenic deletion in *PTPN11* p. E90-Q533 from nevus spilus. (**i**) Protein structure modeling of SHP2, the protein product of *PTPN11,* visualizing the location of the phosphatase and the two SH2 domains. (**j**) Amino acid V428 (green) resides within the WPD-loop of the phosphatase domain, and the P-loop is shown in red, with catalytic C459 in yellow sticks. (**k**) The V428L variant is likely to cause steric clashes and push the P-loop, decreasing the catalytic activity of the phosphatase domain. NGS, next-generation sequencing; PTP, protein tyrosine phosphatase.

DNA extracted directly from the affected skin of nine patients was sequenced using deep-targeted panel sequencing and analyzed with a bioinformatics pipeline optimized for detection of low allele loads, as previously published (Al-Olabi et al., 2018b). We discover here mosaic variants in gene *PTPN11*, which encodes protein SHP2, in eight of the nine patients, at allele loads between 2 and 22% (Figure 2a‒e and Table 1). SHP2 is a ubiquitously expressed nonreceptor protein tyrosine phosphatase actively targeted as a potential therapy for RAS‒MAPK‒driven oncogenic signaling (Chen et al., 2016). Variants were confirmed in affected tissues by Sanger sequencing and were undetected in leukocyte DNA where this was available (Table 1).

Two of the mosaic missense variants (NM_002834.4 c.1381G>A, p.[A461T] and c. 1403C>T, p.[T468M]) have previously been reported to cause Noonan syndrome with lentigines (previously LEOPARD syndrome) (Digilio et al., 2002) as germline mutations, and have also been reported as postnatal somatic mutations in melanoma samples (Forbes et al., 2015). Structural analysis of these two variants has previously shown that although they lead to loss of function at the level of the phosphatase, they increase the affinity of SHP2 for upstream activators, overall resulting in MAPK pathway activation (Yu et al., 2013). The third variant was a large mosaic intragenic deletion (NM_002834.4 c.268_1599+58del, p.[E90-Q533]del) (Figure 2a and h), which to our knowledge is previously unreported, removing the C-SH2 and phosphatase domains, with a predicted loss of function at the level of the phosphatase. A fourth variant also affecting the protein tyrosine phosphatase domain (NM_002834.4 c.1282G>C, p.[V428L]) was a previously unreported missense, identified independently by targeted next-generation sequencing and predicted pathogenic in silico on the basis of SIFT and PolyPhen scores of 0 and 0.93, respectively.

Protein structure modeling of p.(V428L) predicted that this amino acid substitution would cause effects similar to those of the two missense variants already modeled, whereby steric clashes would push the P-loop out of its normal configuration and lead to decreased catalytic activity with increased ligand binding (Figure 2i‒k). The potential mechanism of action of the large intragenic deletion is intriguing and not yet known. Due to ethical constraints of repeat research biopsy in children, it has not been possible to model melanocytes from the affected patient, and specific deletions of this size are technically very difficult to induce in a cell line. Therefore, sporadic melanoma is likely to be a better source of tissue for the study of the signaling pathway effects of *PTPN11* deletions going forward. Given the similarity of the cutaneous phenotypes, we would however hypothesize that the effect of this particular intragenic deletion on MAPK pathway signaling is similar to that of the missense mutations.

Development of two primary melanomas within the nevus spilus was seen in patient 6 carrying the mosaic p.(T468M) variant. Deep-targeted sequencing of melanoma tissue in parallel with the background congenital nevus spilus showed a 4% allele load in the background nevus and a 60% allele load in the superimposed melanoma. This importantly confirms the direct link between the congenital mosaic mutation and melanomagenesis, and confirms *PTPN11* as the oncogenic driver in this case (Figure 2f and g). Given this, and having found the mosaic intragenic deletion in patient 8, we then hypothesized that heterozygous deletions involving *PTPN11* may be unsuspected drivers in sporadic melanoma. To test this hypothesis, deletions involving *PTPN11* were specifically looked for on sequencing data from 345 melanoma tumor samples from the Leeds melanoma cohort. Deletions involving the whole of *PTPN11* were found in 8 of 345 samples, of which six had undergone sequencing for *NRAS* and *BRAF* driver mutations, four of those six were wild type for both and two of the six had a *BRAF* p.(V600E) mutation. Although these data are preliminary, this is a tantalizing suggestion that heterozygous deletions of *PTPN11* may constitute a previously unreported group of potential driver mutations in melanoma.

**Figure 3:**
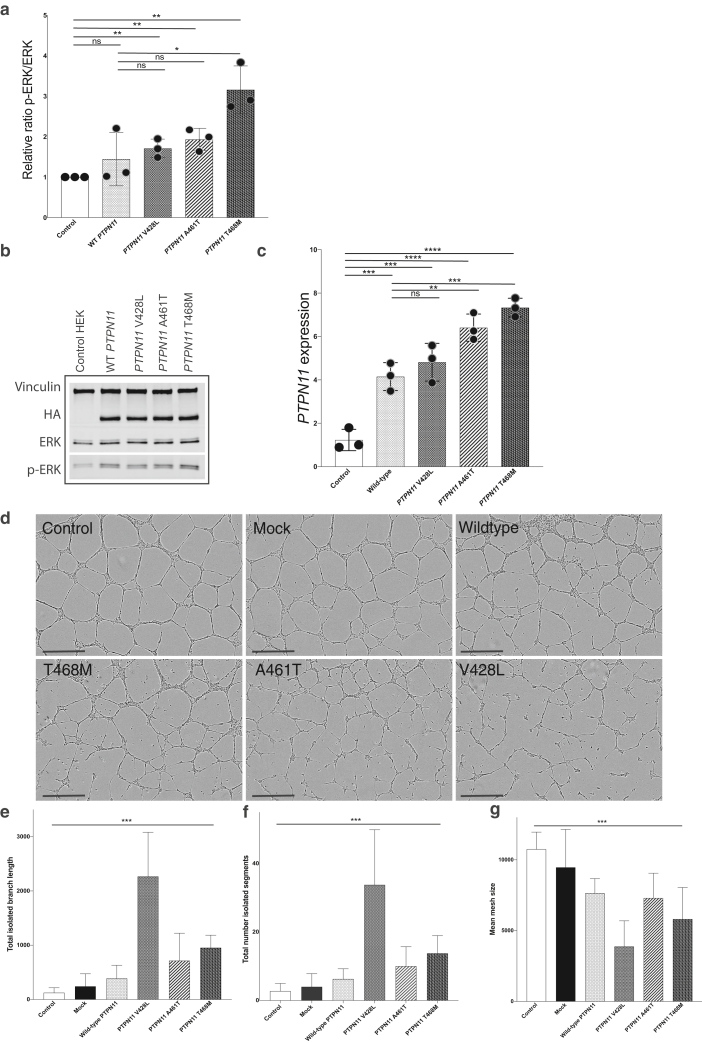
***PTPN11* variant cell models reveal MAPK pathway activation in HEK293 cells and disruption of angiogenesis in a HUVEC primary endothelial cell line.** (**a**) *PTPN11* mRNA expression is significantly increased in WT and all *PTPN11*-variant cells compared with that in untransfected controls, and in *PTPN11*^T468M^ and *PTPN11*^A461T^ variants, *PTPN11* mRNA expression is significantly increased compared with WT overexpression (mean with SD of samples in quadruplicate). (**b, c**) Expression of phosphorylated ERK is significantly increased in all *PTPN11*-variant cells but not in WT cells, compared with that in untransfected controls, and *PTPN11*^T468M^-induced ERK phosphorylation is further significantly increased compared with the WT (mean with SD of samples in triplicate). (**d**) Still images from IncuCyte live-cell analysis during endothelial cell tube formation in vitro show significantly disrupted angiogenesis in HUVEC lines transfected with variant *PTPN11* relative to that in WT and untransfected and mock-transfected cells. Bar = 500 ‒m. (**e‒g**) Quantification of angiogenesis assay (error bars ± SEM) shows a significant difference in variance in (**e**) mean total isolated branch length, (**f**) mean number of isolated segments, and (**g**) mean mesh size. See also Supplementary Movie S1. ERK, extracellular signal‒regulated kinase; HEK293, human embryonic kidney 293; HUVEC, human umbilical vein endothelial cell; ns, not significant; WT, wild type.

**Supplementary Figure S1:**
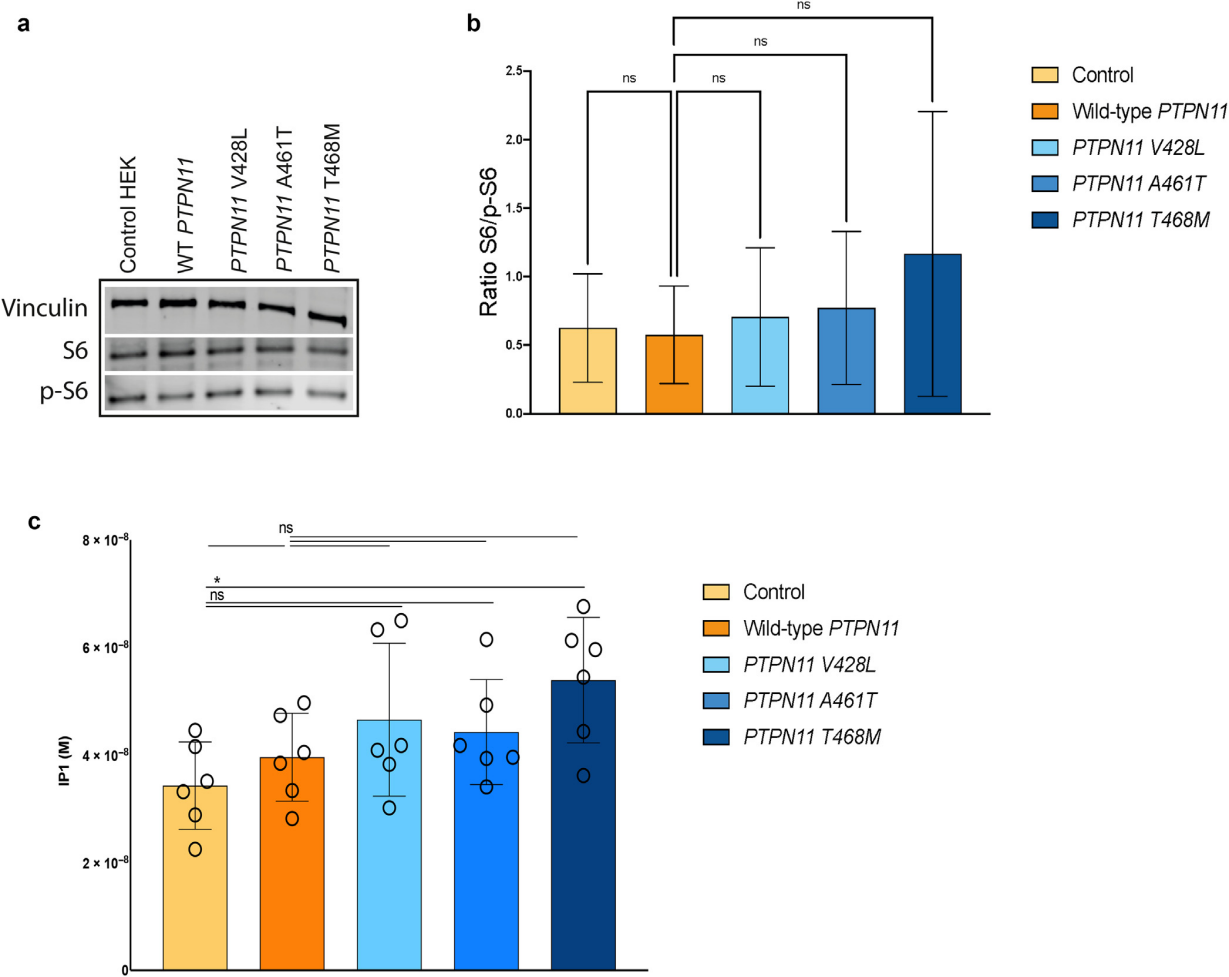
***PTPN11* variant cell models reveal no significant effect on PI3K‒Akt‒mTOR pathway activation or in baseline intracellular calcium in HEK293 cells.** (**a, b**) Western blotting showed no statistical change in the protein expression of phosphorylated S6 in all the three stable cells lines expressing *PTPN11* variants when compared with the overexpression of wildtype *PTPN11* or control HEK293 cells (mean of densitometric analysis is shown with SD, samples are in triplicate). (**a**) IP1 assay did not show any statistically significant variation in IP1 in *PTPN11* variants when compared with that in the control cell lines (mean shown with SD of six technical replicates is shown). Akt, protein kinase B; HEK293, human embryonic kidney 293; IP1, inositol monophosphate; ns, not significant; p-S6, phosphorylated S6.

To further investigate the functional properties of the *PTPN11* missense variants, we generated stable overexpression cell lines using *PTPN11* cDNA constructs for wild type and all the three missense variants affecting the PTP domain. Both wild-type and variant constructs led to significantly increased mRNA expression of *PTPN11* compared with that of untransfected lines, but this was significantly more for variants p.(A461T) and p.(T468M) than for wild type. Furthermore, variant constructs but not wild type led to significant activation of the MAPK pathway compared with those of untransfected cell lines, with additionally significantly increased activation for variant p.(T468M) over the wild type (Figure 3a‒c). In parallel, given that there was a degree of overlap of effects of wild-type overexpression and variant overexpression, we were keen to test whether other known major signaling pathways could be affected by the signaling. We therefore assessed downstream activation of the PI3K‒protein kinase B‒mTOR pathway by pS6 activation as well as intracellular calcium signaling using inositol monophosphate levels. No statistically significant differences between wild-type and *PTPN11* cells were observed in either of these pathways, confirming that MAPK activation appears to be the mechanism of action at least in human embryonic kidney 293 cells (Supplementary Figure S1).

We then proceeded to model the functional effects of the variants using a disease-relevant primary endothelial cell line and a vascular tube-forming assay*.* Transient transfection of human umbilical vein endothelial cells was undertaken, and angiogenesis assessed on a Geltrex matrix using well-established readouts from previous studies of vascular malformations (Al-Olabi et al., 2018a; Arnaoutova and Kleinman, 2010; Takahashi et al., 2021). All three *PTPN11* variants significantly disrupted vascular endothelial angiogenesis compared with those of control lines using multiple measurements (Figure 3d‒g). Wild-type *PTPN11* overexpression had a significantly disruptive effect itself but to a lesser degree than that of the variants (Figure 3d‒g). Time-lapse IncuCyte imaging of angiogenesis over 24 hours showed marked impairment in tube formation ability by the variant human umbilical vein endothelial cells, clearly visible as variation in size and solidity of structures compared with those of controls (Supplementary Movie S1).

## Discussion

Our discovery of *PTPN11* mosaicism across a spectrum of previously distinct clinical diagnoses causing pigmentary and/or vascular abnormalities of the skin and musculoskeletal, neurological, and ophthalmological abnormalities has important potential clinical implications. First, it is well-established that mosaic mutations can be passed on to offspring as germline heterozygous disease, for example, epidermal naevi with keratin 1 gene *K1* and keratin 10 gene *K10* mutations passed on as epidermolytic ichthyosis (Nomura et al., 2001; Paller et al., 1994) or mosaic NF1 being passed down as germline NF1 (Rasmussen et al., 1998; Tinschert et al., 2000; Zlotogora, 1993). This is theoretically a possibility in any mosaic condition where the gametes are affected and the mosaic variant is not lethal in a germline heterozygous state (Kinsler et al., 2020). This will be true whether or not such transmission has been described in the literature because in cases where the mosaic and germline heterozygous disease states are not phenotypically similar, the two may not have been considered to be connected in the minds of physicians. For example, identical *FGFR3* mutations in mosaic form produce epidermal nevi (MIM162900), whereas in the germline, they cause thanatophoric dysplasia (MIM 187600). As it is not possible to predict, or usually feasible to test, whether gametes are affected in any one individual, the fact that at least two of the *PTPN11* variants described in this study are known to be compatible with life in the germline means that mosaic patients should be counseled that there is an unknown risk of having offspring with Noonan syndrome with lentigines. This is a serious germline condition commonly including cardiac and large blood vessel abnormalities, which can reduce life expectancy as well as cause deafness, developmental delay, and abnormal genitalia. Unfortunately, the risk of transmission to future generations is currently unpredictable because of the demonstrable lack of correlation between mutant allele load and tissue histopathological abnormality in other mosaic conditions (Doucet et al., 2016) and the asymmetry of cell division within somatic tissues (Ju et al., 2017), which could give rise to selective advantage or loss of the mutant allele in gametes. Furthermore, rare insights from mosaicism in sperm donors suggest that the risk can be very substantial when dealing with variants that are compatible with survival in a germline state (Ejerskov et al., 2016). Hence, a molecular diagnosis in this mosaic condition has implications for future genetic counseling.

Secondly, a molecular diagnosis confirms that these specific patients with *PTPN11* mosaic nevus spilus are at risk of melanoma. PPV and nevus spilus as clinical diagnoses are already known to predispose to melanoma; however, this risk has not previously been stratified by the causative genotype of the underlying condition, and it is possible that different genotypes will have different risks of melanoma. Specifically, nevus spilus can also be caused by variants in *HRAS* (Sarin et al., 2013), but *HRAS* is a rare driver of melanoma (Chin et al., 2006; Forbes et al., 2015) and is less frequently found mutated in melanoma than *PTPN11* (Forbes et al., 2015). In this study, one older adult developed two primary melanomas within the same nevus spilus, with an increased allele load indicative of the clonal expansion of *PTPN11* mosaic melanocytes into melanoma. *PTPN11* missense and short insertion‒deletion variants have been reported in 58 of 1,030 (6%) melanoma samples in the COSMIC database (including two of the variants we describe in this study), and *PTPN11* has recently been implicated in the pathogenesis of *BRAF*-wild-type melanoma (Hill et al., 2019). The finding of deletions involving the whole of *PTPN11* in 8 of 346 melanoma samples in this study suggests an interesting new perspective on the role of *PTPN11* in driving melanoma. Prospective studies with functional analysis of *PTPN11* deletions in melanoma will however be needed to establish whether this is correct.

Functional work on the missense variants here raises some interesting questions about defining pathogenicity at the functional level. When dealing with loss-of-function variants, the gold standard of pathogenicity is usually considered to be a significant difference in biological effect between wild type and variant. With gain-of-function oncogenic mutations, however, as we have here, it is known that wild-type overexpression can lead to the same effects as a variant (Gracilla et al., 2020; Hortal et al., 2022; Zhou et al., 2016). In our results, we show this clearly in the MAPK activating effects of wild-type overexpression, albeit to a significantly lower level than with certain of the missense variants. We also see differences in the strength of effect of the different missenses in the context of signaling pathway measurement versus the angiogenesis assay, which may reflect cell-type specific variation. Most importantly, however, overexpression of variants is not having a nonspecific overactivating effect on cell signaling, as shown by a lack of any effect on the protein kinase B‒mTOR and intracellular calcium signaling pathways. MAPK-driven abnormalities of developmental biology and postnatal cellular function are therefore the highly likely mechanisms of action in this spectrum of disease.

These findings also contribute to our understanding of embryology. Classic developmental biology teaches us that all cells and tissues develop from the three germ layers: ectoderm, mesoderm, and endoderm, with the neural crest often considered as a fourth germ layer. In recent years, our understanding of the details of this developmental lineage segregation has been challenged by single-cell and lineage-tracing technologies (McKinley et al., 2020; Olsson et al., 2016; Peng et al., 2019). With these powerful approaches, novel cell lineages have been identified (Grün et al., 2015), and the existence of progenitor cells with the potential to make binary cell fate choices have redefined established cell-fate hierarchies (Olsson et al., 2016). This emerging knowledge has increased our understanding of lineage commitment in early development but has not yet been applied to explain human disease phenotypes. Two recent discoveries have challenged the dogma that vascular endothelial cells and melanocytes develop from different germ layers: endothelial cells from the mesoderm and melanocytes from the ectodermal neural crest (Adameyko et al., 2009; Erickson and Goins, 1995). The first study reported that individuals diagnosed with PPV types I, II, and V, also known as cesioflammea and cesiomarmorata types, are clonally mosaic for a *GNA11* or *GNAQ* variant (Sliepka et al., 2019; Thomas et al., 2016), and that individuals affected by only one or other type of lesion had the same genetic cause (Shirley et al., 2013; Thomas et al., 2016). These clinical observations led us to hypothesize that a common embryonic precursor for melanocytic and vascular cells might exist before they adopt separate lineages (Thomas et al., 2016). The second was the description of pigmentary and vascular abnormalities in individuals with an entirely distinct mosaic disorder congenital melanocytic nevus syndrome caused by *NRAS* (Al-Olabi et al., 2018b) or *BRAF* (Etchevers et al., 2018) mosaic variants. The discovery here of clonal *PTPN11* variants producing another phenotypically distinct type of PPV adds further weight to the hypothesis. Isolation/identification of mutant cells from each lesion type has not yet been possible alongside gene discovery because of ethical constraints related to extensive sampling in children for research purposes, but future dedicated studies will be able to answer this question.

In this study, we identify mosaic *PTPN11* variants causing a spectrum of rare pigmentary and vascular syndromes with associated multisystem abnormalities and a predisposition to malignancy. As at least two of the four variants have been shown to be compatible with life in a germline state, mosaic *PTPN11* variant carriers are at theoretically increased risk of having children with Noonan syndrome with multiple lentigines. Identification of a mosaic intragenic deletion causing the same phenotype as known driver missense variants has led to the identification of deletions involving *PTPN11* in somatic form in a melanoma cohort. This will potentially lead to a broadening of the understanding of the impact of *PTPN11* on malignancy more widely. These new insights should improve our clinical management of *PTPN11*-driven disease.

## Materials and Methods

For methods of DNA extraction, next-generation sequencing, quantitative real time PCR, and western blotting, see Supplementary Materials and Methods.

### Patient cohorts

Nine patients with either PPV (spilorosea or type III) (n = 4), speckled lentiginous nevus syndrome (n = 1), nevus spilus (n = 3), and capillary malformation (n = 1), most with associated abnormalities in other organ systems (Table 1), were recruited in a multicentre international collaboration. Seven patients with classical PPV type III/spilorosea were initially recruited alongside patients with congenital nevus spilus (and no other cutaneous findings) because it was felt that these were likely to be on the same genotypic spectrum. In a separate study, two patients were found to have the same genotypic diagnosis, including a single patient with a large capillary malformation and no other cutaneous findings; these were therefore included.

### Ethics Approval and Consent to Participate

All participants gave written informed consent as part of ethically approved studies. The study was approved by the London Bloomsbury Research Ethics Committee of Great Ormond Street Hospital/UCL Institute of Child Health (London, United Kingdom).

### Statistics

For quantitative real time PCR data, one-way ANOVA was performed using either the wild type or the control as the independent variable (Prism, version 7.0, Graphpad Software, San Diego, CA). For angiogenesis assay data, one-way ANOVA was performed using the cell type as the independent variable, which had six groups: control cells, mock transfected, wild type *PTPN11*, *PTPN11*^T468M^, *PTPN11*^A461T^, and *PTPN11*^V428L^. Means of 20 standardized measurable outcomes of the assay were generated, with correction for multiple testing reducing the significance level to *P* < 0.0025. For western blot quantification, statistical significance was determined using a two-tailed Student’s *t*-test (Prism, version 7.0, GraphPad Software). Throughout, statistically significant values are indicated by a single asterisk (*P* < 0.05), a double asterisk *P* < 0.01, a triple asterisk (*P* < 0.001), or a quadruple asterisk (*P* < 0.0001).

### Generation of stable cell lines

Human embryonic kidney 293 cells were cultured in DMEM 10% fetal bovine serum as per the manufacturer’s instructions. *PTPN11* was sequenced in cell lines to verify wild-type sequences. Human *PTPN11*^*WT*^ (NM_002834.5)*, PTPN11*^*G1282C*^*, PTPN11*^*G1381A*^, and *PTPN11*^*C1403T*^ cDNAs were synthesized and cloned into a pcDNA3.1+ N-HA plasmid, fused in frame at their N-terminus with an HA tag (Genscript Biotech, Piscataway, NJ). Luciferase ORF was excised from pLenti PGK V5-LUC Neo (21471, Addgene, Watertown, MA) by SalI and XbaI combined restriction digestion, and HA-tagged *PTPN11* cDNAs were amplified and cloned into the digested pLenti-vector using the In-Fusion HD Cloning kit (638947, Takara Bio, Shiga, Japan), following the on-line primer design tool and the manufacturer's instructions.

Transduction of human embryonic kidney 293T cells with pLenti PGK *PTPN11*^*WT*^*, PTPN11*^*G1282C*^*, PTPN11*^*G1381A*^*,* or *PTPN11*^*C1403T*^ was done in addition to pCMV-VSVG and delta-8.2 (Addgene) lentiviral plasmids using Lipofectamine 2000‒generated lentiviral particles used to infect human embryonic kidney 293 target cells using 8 μg/ml polybrene to enhance efficiency. Stable cell lines were selected using G418. All variants were verified in cell lines with Sanger Sequencing.

### Endothelial Tube Formation Assay

Passage 0 human umbilical vein endothelial cells (catalog number C0035C, Thermo Fisher Scientific, Waltham, MA) were thawed and maintained in endothelial cell media (EGM2, catalog number CC-3162, Lonza, Basel, Switzerland). At postnatal day 4, cells were seeded in T75 tissue culture flasks. The following day, cells were transfected with either variant or wild-type plasmid using Lipofectamine LTX with plus reagent (catalog number 15338100, Thermo Fisher Scientific). The transfection protocol was optimized as follows: 12 μg of plasmid DNA and 12 μl of Plus reagent were diluted up to 750 μl in Opti-MEM I Reduced Serum Medium (catalog 31985062) and incubated at room temperature for 5 minutes and then added to 42 μl of Lipofectamine LTX diluted up to 750 ul in Opti-MEM I Reduced Serum Medium. Plasmid solutions were incubated for 20 minutes at room temperature to allow complexes to form. Complexes (1,500 μl) were then added to each flask, and cells were incubated at 37 °C in a 5% carbon dioxide incubator for 48 hours. Transfected and untreated cells were then trypsinized and counted, and 5 × 104 cells were seeded into individual wells of two separate 24-well plates coated with GF–reduced Geltrex (catalog A1413202) at 100 μl/well. One plate was further incubated for 20 hours. Thirty minutes before the end of the incubation period, the cells of one 24-well plate cells were treated with 2 μg/ml (0.8 μl/well) calcein AM (catalog C3099). Tube formation observed at the 6-hour time point was imaged with a ×5 objective lens of an Olympus IX71 inverted fluorescence and bright field microscope using HCImage software. The degree of tube formation was assessed by measuring all aspects of tubule, node, and mesh growth in quadruplicate using randomly chosen fields from each well using the angiogenesis analyzer for ImageJ (http://image.bio.methods.free.fr/ImageJ/?Angiogenesis-Analyzer-for-ImageJ). In parallel, the second 24-well plate was analyzed using the IncuCyte live-cell analysis system and set up to acquire X4 phase contrast images at a scanning interval of 60 minutes for 48 hours.

### Clinical images

Patients and/or parents/guardians provided written consent for the publication of images.

### Data Availability Statement

No datasets were generated or analyzed during this study.

## ORCIDs

Satyamaanasa Polubothu: http://orcid.org/0000-0001-7195-5670

Nicole Bender: http://orcid.org/0000-0003-3931-9839

Siobhan Muthiah: http://orcid.org/0000-0003-0283-2719

Davide Zecchin: http://orcid.org/0000-0002-4784-0336

Charalambos Demetriou: http://orcid.org/0000-0001-6630-1322

Sara Barberan Martin: http://orcid.org/0000-0003-0142-4078

Sony Malhotra: http://orcid.org/0000-0002-3165-9081

Jana Travnickova: http://orcid.org/0000-0002-8339-9162

Zhiqiang Zeng: http://orcid.org/0000-0002-9533-3347

Markus Böhm: http://orcid.org/0000-0001-7338-7734

Catherine Cottrell: http://orcid.org/0000-0002-2974-0235

Sebastien Barbarot: http://orcid.org/0000-0002-6629-9100

Olivia Davies: http://orcid.org/0000-0001-5428-7279

Eulalia Baselga: http://orcid.org/0000-0003-1086-8439

Nigel Burrows: http://orcid.org/0000-0002-1090-8261

Virginie Carmignac: http://orcid.org/0000-0001-8802-6448

Joey Mark Diaz: http://orcid.org/0000-0002-3670-2288

Christine Fink: http://orcid.org/0000-0002-3875-6415

Holger Haenssle: http://orcid.org/0000-0001-7255-3104

Rudolf Happle: http://orcid.org/0000-0002-6338-1766

Mark Harland: http://orcid.org/0000-0002-0077-9742

Jacqueline Majerowski: http://orcid.org/0000-0002-4966-9990

Pierre Vabres: http://orcid.org/0000-0001-8693-3183

Marie Vincent: http://orcid.org/0000-0003-1010-5618

Julia Newton-Bishop: http://orcid.org/0000-0001-9147-6802

D. Timothy Bishop: http://orcid.org/0000-0002-8752-8785

Dawn Siegel: http://orcid.org/0000-0001-6546-2693

E. Elizabeth Patton: http://orcid.org/0000-0002-2570-0834

Maya Topf: http://orcid.org/0000-0002-8185-1215

Neil Rajan: http://orcid.org/0000-0002-5850-5680

Beth Drolet: http://orcid.org/0000-0002-0844-7195

Veronica A Kinsler: http://orcid.org/0000-0001-6256-327X

## Conflict of Interest

BD is a Venthera consultant and cofounder of Arkayli. The remaining authors state no conflict of interest.

## Figures and Tables

**Supplementary Table S1:** Sequencing Primers for *PTPN11* Sanger Sequencing

Variant	Forward Primer	Reverse Primer
Variant 1 Chr12:112926248G>A	CAAAGCCCTATGCTTTTTGC	CACCAGATGACCCACCTTTC
Variant 2 Chr12:112926270C>T	CAAAGCCCTATGCTTTTTGC	CACCAGATGACCCACCTTTC
Variant 3 Chr12:112888251-112927037	AAAATCCGACGTGGAAGATG	CCGTCATGCATTTCTGACAC
Variant 4		

**Supplementary Table S2:** Taqman Probes

Gene	Reference Number
*PTPN11*	Hs06636344_g1
*GAPDH*	Hs02786624_g1

**Supplementary Table S3:** Antibodies, Source, and Dilution

Antibody	Source	Catalog Number	Dilution
Vinculin	Mouse	Cell Signaling Technology #4650S	1:5,000
ERK	Mouse	Cell Signaling Technology #9107	1:1,000
Phosphorylated ERK	Rabbit	Cell Signaling Technology #9101	1:1,000
HA.11 clone 16B12	Mouse	BioLegend #901513	1:1,000
S6	Mouse	Cell Signaling Technology #2317	1:5,000
Phosphorylated S6	Rabbit	Cell Signaling Technology #2211	1:1,000

Abbreviation: ERK, extracellular signal‒regulated kinase.

## Supplementary Materials and Methods

### DNA extraction

DNA was extracted directly from affected skin samples (DNeasy Blood and Tissue Kit, Qiagen, Manchester, United Kingdom).

### Next-generation sequencing and variant calling

The affected skin DNA from four patients was sequenced on a targeted next-generation sequencing panel using a SureSeq Custom panel of 250 candidate genes of interest. DNA library preparation was performed manually using the Agilent SureSelect XT Reagent Kit as per the manufacturer’s instructions. Pooled libraries were diluted to 1.4 picomolar, spiked with 1% PhiX DNA (Illumina, San Diego, CA), and sequenced on a Nextseq 3000 instrument (Illumina) according to the manufacturer’s recommendations, for paired-end 150-bp reads. Sequence alignment to the human reference genome (UCSC hg19), and variant calling and annotation were performed with our in-house mosaic pipeline, as described previously (https://genome.ucsc.edu/). Resultant .vcf files were further analyzed using Ingenuity variant analysis.

For a further two patients, 100 ng of DNA was amplified using primers specific to *PTPN11* hotspots (primers in Supplementary Table S1) using standard PCR methods and PrimeSTAR GXL DNA Polymerase (Takara Bio, Shiga, Japan). PCR amplicons were purified and prepared manually using Nextera XT DNA library kit (Illumina). Libraries were sequenced on a MiSeq instrument with a 300-cycle MiSeq Reagent Kit, version 2 (Illumina), and paired-end sequencing reactions of 150-bp reads. Sequence alignment to the human reference genome (UCSC hg19) and variant calling and annotation were performed with our in-house pipeline.

All variants were confirmed by Sanger Sequencing, in parallel with DNA extracted from the blood, where available.

### Quantitative real-time PCR

A total of 1 μg of total RNA was reverse transcribed, and quantitative Real-Time PCR was performed, using Taqman probes (Supplementary Table S2) and the QuantStudio 7 Flex System (Applied Biosystems, Waltham, MA). Samples were analyzed in biological triplicates and technical quadruplicates using a 384-well plate. Relative fold change to the housekeeping gene *GAPDH* was calculated by the ΔΔCT method.

### Western Blotting

Protein lysate was quantified using the Bradford protein assay, and 20 μg of each sample was run on a 10% Bis-Tris protein gel. Fluorescent polyvinylidene fluoride membrane was activated in methanol before transfer using a wet transfer reservoir. The membrane was washed three times in Tris Buffered Saline-Tween and then blocked using 5% cold water fish skin at room temperature for 1 hour. Membranes were then incubated in primary antibody in 5% albumin overnight at 4 °C (see Supplementary Table S3 for source and dilution). Membranes were washed three times in Tris Buffered Saline-Tween and then incubated with secondary conjugates compatible with infrared detection at 700 and 800 nm, and membranes were scanned using the Odyssey Infrared Imaging System (Odyssey, LI-COR Bioscience, Lincoln, NE). The intensity of bands was measured and quantified using ImageJ software.

### Inositol monophosphate assay

Intracellular concentrations of inositol monophosphate, a downstream metabolite of inositol trisphosphate, which is a key mediator of intracellular calcium signal, were quantified in human embryonic kidney 293 cells (control and stable expression of wild-type *PTPN11* and *PTPN11* variants) using HTRF-IP-One kit (Cisbio Bioassays, Codolet, France) as per the manufacturer's instructions. Briefly, cells were trypsinized, and cell pellets were resuspended in complete medium and transferred to a 384-well microtitre plate at a density of 50,000 cells/7 μl in each well, and a total of six wells were used as technical replicates for each experimental condition. A total of 7 μl of stimulation buffer was then added to each well. After 90 minutes of incubation at 37 ºC, 3 μl of inositol monophosphate-d2 conjugate and 3 μl of europium cryptate-labeled anti-inositol monophosphate antibody dissolved in lysis buffer were added to the cells. After incubation in the dark for 1 hour at room temperature, fluorescence was sequentially measured at 620 and 665 nm in every well using the Tecan Spark plate reader.

## Supplementary Material

Supplementary Movie S1 Endothelial cell tube formation in wildtype and PTPN11 variant cells. This movie is associated with Figure 3. Time-lapse video recorded with the IncuCyte live-cell analysis system showing endothelial cell tube formation in control HUVEC lines, mock transfection, and transfection with wild-type and mutant PTPN11 constructs. Disordered formation of vascular networks is observed in mutant PTPN11 constructs and to a lesser extent in wild-type constructs than in the controls. HUVEC, human umbilical vein endothelial cell.

## References

[bib1] Adameyko I., Lallemend F., Aquino J.B., Pereira J.A., Topilko P., Müller T. (2009). Schwann cell precursors from nerve innervation are a cellular origin of melanocytes in skin. Cell.

[bib2] Al-Olabi L., Polubothu S., Dowsett K., Andrews K.A., Stadnik P., Joseph A.P. (2018). Mosaic RAS/MAPK variants cause sporadic vascular malformations which respond to targeted therapy. J Clin Invest.

[bib3] Al-Olabi L., Polubothu S., Dowsett K., Andrews K.A., Stadnik P., Joseph A.P. (2018). Mosaic RAS/MAPK variants cause sporadic vascular malformations which respond to targeted therapy. J Clin Invest.

[bib4] Arnaoutova I., Kleinman H.K. (2010). In vitro angiogenesis: endothelial cell tube formation on gelled basement membrane extract. Nat Protoc.

[bib5] Borrego L., Hernandez Santana J., Baez O., Hernandez Hernandez B. (1994). Naevus spilus as a precursor of cutaneous melanoma: report of a case and literature review. Clin Exp Dermatol.

[bib6] Chen Y.N., LaMarche M.J., Chan H.M., Fekkes P., Garcia-Fortanet J., Acker M.G. (2016). Allosteric inhibition of SHP2 phosphatase inhibits cancers driven by receptor tyrosine kinases. Nature.

[bib7] Chin L., Garraway L.A., Fisher D.E. (2006). Malignant melanoma: genetics and therapeutics in the genomic era. Genes Dev.

[bib8] Digilio M.C., Conti E., Sarkozy A., Mingarelli R., Dottorini T., Marino B. (2002). Grouping of multiple-lentigines/Leopard and Noonan syndromes on the PTPN11 gene. Am J Hum Genet.

[bib9] Doucet M.E., Bloomhardt H.M., Moroz K., Lindhurst M.J., Biesecker L.G. (2016). Lack of mutation-histopathology correlation in a patient with Proteus syndrome. Am J Med Genet A.

[bib10] Ejerskov C., Farholt S., Skovby F., Vestergaard E.M., Haagerup A. (2016). Clinical presentations of 23 half-siblings from a mosaic neurofibromatosis type 1 sperm donor. Clin Genet.

[bib11] Erickson C.A., Goins T.L. (1995). Avian neural crest cells can migrate in the dorsolateral path only if they are specified as melanocytes. Development.

[bib12] Etchevers H.C., Rose C., Kahle B., Vorbringer H., Fina F., Heux P. (2018). Giant congenital melanocytic nevus with vascular malformation and epidermal cysts associated with a somatic activating mutation in BRAF. Pigment Cell Melanoma Res.

[bib13] Fink C., Happle R., Enk A., Haenssle H.A. (2016). Phacomatosis spilorosea: visual diagnosis and associated pathologies of a rare entity. J Eur Acad Dermatol Venereol.

[bib14] Forbes S.A., Beare D., Gunasekaran P., Leung K., Bindal N., Boutselakis H. (2015). COSMIC: exploring the world's knowledge of somatic mutations in human cancer. Nucleic Acids Res.

[bib15] Gracilla D.E., Korla P.K., Lai M.T., Chiang A.J., Liou W.S., Sheu J.J.-C. (2020). Overexpression of wild type or a Q311E mutant MB21D2 promotes a pro-oncogenic phenotype in HNSCC. Mol Oncol.

[bib16] Groesser L., Herschberger E., Ruetten A., Ruivenkamp C., Lopriore E., Zutt M. (2012). Postzygotic HRAS and KRAS mutations cause nevus sebaceous and Schimmelpenning syndrome. Nat Genet.

[bib17] Grün D., Lyubimova A., Kester L., Wiebrands K., Basak O., Sasaki N. (2015). Single-cell messenger RNA sequencing reveals rare intestinal cell types. Nature.

[bib18] Happle R. (2005). Phacomatosis pigmentovascularis revisited and reclassified. Arch Dermatol.

[bib19] Hill K.S., Roberts E.R., Wang X., Marin E., Park T.D., Son S. (2019). PTPN11 plays oncogenic roles and is a therapeutic target for BRAF wild-type melanomas. Mol Cancer Res.

[bib20] Hortal A.M., Oeste C.L., Cifuentes C., Alcoceba M., Fernández-Pisonero I., Clavaín L. (2022). Overexpression of wild type RRAS2, without oncogenic mutations, drives chronic lymphocytic leukemia. Mol Cancer.

[bib21] Ju Y.S., Martincorena I., Gerstung M., Petljak M., Alexandrov L.B., Rahbari R. (2017). Somatic mutations reveal asymmetric cellular dynamics in the early human embryo. Nature.

[bib22] Keppler-Noreuil K.M., Rios J.J., Parker V.E., Semple R.K., Lindhurst M.J., Sapp J.C. (2015). PIK3CA-related overgrowth spectrum (PROS): diagnostic and testing eligibility criteria, differential diagnosis, and evaluation. Am J Med Genet A.

[bib23] Keppler-Noreuil K.M., Sapp J.C., Lindhurst M.J., Darling T.N., Burton-Akright J., Bagheri M. (2019). Pharmacodynamic study of Miransertib in individuals with Proteus syndrome. Am J Hum Genet.

[bib24] Kinsler V.A., Boccara O., Fraitag S., Torrelo A., Vabres P., Diociauti A. (2020). Mosaic abnormalities of the skin - review and guidelines from the European Reference Network for rare skin diseases (ERN-Skin). Br J Dermatol.

[bib25] Lindhurst M.J., Sapp J.C., Teer J.K., Johnston J.J., Finn E.M., Peters K. (2011). A mosaic activating mutation in AKT1 associated with the Proteus syndrome. N Engl J Med.

[bib26] Manganoni A.M., Pavoni L., Farisoglio C., Sereni E., Calzavara-Pinton P. (2012). Report of 27 cases of naevus spilus in 2134 patients with melanoma: is naevus spilus a risk marker of cutaneous melanoma?. J Eur Acad Dermatol Venereol.

[bib27] Martincorena I., Campbell P.J. (2015). Somatic mutation in cancer and normal cells. Science.

[bib28] McKinley K.L., Castillo-Azofeifa D., Klein O.D. (2020). Tools and concepts for interrogating and defining cellular identity. Cell Stem Cell.

[bib29] Nomura K., Umeki K., Hatayama I., Kuronuma T. (2001). Phenotypic heterogeneity in bullous congenital ichthyosiform erythroderma: possible somatic mosaicism for keratin gene mutation in the mildly affected mother of the proband. Arch Dermatol.

[bib30] Olsson A., Venkatasubramanian M., Chaudhri V.K., Aronow B.J., Salomonis N., Singh H. (2016). Single-cell analysis of mixed-lineage states leading to a binary cell fate choice. Nature.

[bib31] Ota M., Kawamura T., Ito N. (1947). Phakomatosis pigmentovascularis. Jpn J Dermatol.

[bib32] Paller A.S., Syder A.J., Chan Y.M., Yu Q.C., Hutton E., Tadini G. (1994). Genetic and clinical mosaicism in a type of epidermal nevus. N Engl J Med.

[bib33] Peng G., Suo S., Cui G., Yu F., Wang R., Chen J. (2019). Molecular architecture of lineage allocation and tissue organization in early mouse embryo. Nature.

[bib34] Rasmussen S.A., Colman S.D., Ho V.T., Abernathy C.R., Arn P.H., Weiss L. (1998). Constitutional and mosaic large NF1 gene deletions in neurofibromatosis type 1. J Med Genet.

[bib35] Riachi M., Polubothu S., Stadnik P., Hughes C., Martin S.B., Charman C.R. (2021). Molecular genetic dissection of inflammatory linear verrucous epidermal naevus leads to successful targeted therapy. J Invest Dermatol.

[bib36] Sarin K.Y., Sun B.K., Bangs C.D., Cherry A., Swetter S.M., Kim J. (2013). Activating HRAS mutation in agminated Spitz nevi arising in a nevus spilus. JAMA Dermatol.

[bib37] Shirley M.D., Tang H., Gallione C.J., Baugher J.D., Frelin L.P., Cohen B. (2013). Sturge-Weber syndrome and port-wine stains caused by somatic mutation in GNAQ. N Engl J Med.

[bib38] Sliepka J.M., McGriff S.C., Rossetti L.Z., Bizargity P., Streff H., Lee Y.S. (2019). GNA11 brain somatic pathogenic variant in an individual with phacomatosis pigmentovascularis. Neurol Genet.

[bib39] Takahashi Y., Kawasaki T., Sato H., Hasegawa Y., Dudek S.M., Ohara O. (2021). Functional roles for CD26/DPP4 in mediating inflammatory responses of pulmonary vascular endothelial cells. Cells.

[bib40] Thomas A.C., Zeng Z., Rivière J.B., O'Shaughnessy R., Al-Olabi L., St-Onge J. (2016). Mosaic activating mutations in GNA11 and GNAQ are associated with phakomatosis pigmentovascularis and extensive dermal melanocytosis. J Invest Dermatol.

[bib41] Tinschert S., Naumann I., Stegmann E., Buske A., Kaufmann D., Thiel G. (2000). Segmental neurofibromatosis is caused by somatic mutation of the neurofibromatosis type 1 (NF1) gene. Eur J Hum Genet.

[bib42] Torchia D. (2013). Framing phacomatosis spilorosea. J Eur Acad Dermatol Venereol.

[bib43] Tsuruta D., Fukai K., Seto M., Fujitani K., Shindo K., Hamada T. (1999). Phakomatosis pigmentovascularis type IIIb associated with Moyamoya disease. Pediatr Dermatol.

[bib44] Venot Q., Blanc T., Rabia S.H., Berteloot L., Ladraa S., Duong J.P. (2018). Targeted therapy in patients with PIK3CA-related overgrowth syndrome. Nature.

[bib45] Vidaurri-de la Cruz H., Happle R. (2006). Two distinct types of speckled lentiginous nevi characterized by macular versus papular speckles. Dermatology.

[bib46] Weinberg J.M., Schutzer P.J., Harris R.M., Tangoren I.A., Sood S., Rudolph R.I. (1998). Melanoma arising in nevus spilus. Cutis.

[bib47] Yu Z.H., Xu J., Walls C.D., Chen L., Zhang S., Zhang R. (2013). Structural and mechanistic insights into Leopard syndrome-associated SHP2 mutations. J Biol Chem.

[bib48] Zhou B., Der C.J., Cox A.D. (2016). The role of wild type RAS isoforms in cancer. Semin Cell Dev Biol.

[bib49] Zlotogora J. (1993). Mutations in von Recklinghausen neurofibromatosis: an hypothesis. Am J Med Genet.

